# Supplementary material for: Optimization of shadow evaporation and oxidation for reproducible quantum Josephson junction circuits
Source: Sci Rep. 2023 Mar 13;13:4174. doi: 10.1038/s41598-023-31003-1 (PMC10011367; doi:10.1038/s41598-023-31003-1)
Supplement: Supplementary file 1 — Supplementary Information. [file 41598_2023_31003_MOESM1_ESM.docx]

**Supplementary Information**

**Optimization of shadow evaporation and oxidation for reproducible quantum Josephson junction circuits**

Dmitry O. Moskalev^1,2^, Evgeniy V. Zikiy^1,2^, Anastasiya A. Pishchimova^1,2^,

Daria A. Ezenkova^1,2^, Nikita S. Smirnov^1^, Anton I. Ivanov^1^, Nikita D. Korshakov^1^,

and Ilya A. Rodionov^1,2,*^

^1^ FMN Laboratory, Bauman Moscow State Technical University, Moscow, 105005, Russia

^2^ Dukhov Automatics Research Institute (VNIIA), Moscow, 127055, Russia

^*^ Electronic mail: irodionov@bmstu.ru

**Different deposition scheme**

We fabricated test 20×20 mm^2^ area chips to evaluate the influence of the surface roughness of thin film bottom electrodes on JJ critical current reproducibility. Test chips contained Josephson junctions of different areas (100×100 nm^2^, 150×200 nm^2^ and 150×600 nm^2^). 2 schemes for the bottom electrode deposition were considered: 25 nm/45º and 15 nm/0º. Critical current variation (*σ/⟨I_c_⟩*) of Josephson junctions for different deposition scheme is shown in Table S1.

**Table S1.** Critical current variation (*σ/⟨I_c_⟩*) of Josephson junctions for different deposition scheme

| **Deposition**  **scheme** | **100×100 nm^2^ JJ area** | **150×200 nm^2^ JJ area** | **150×600 nm^2^ JJ area** |
| --- | --- | --- | --- |
| **25 nm/45º** | 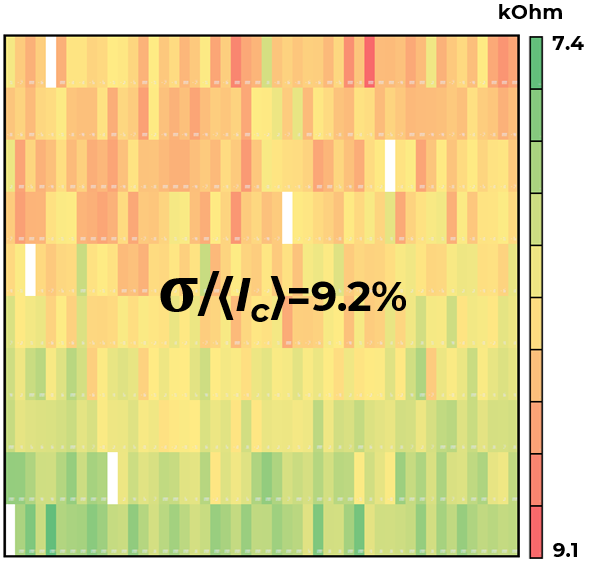 | 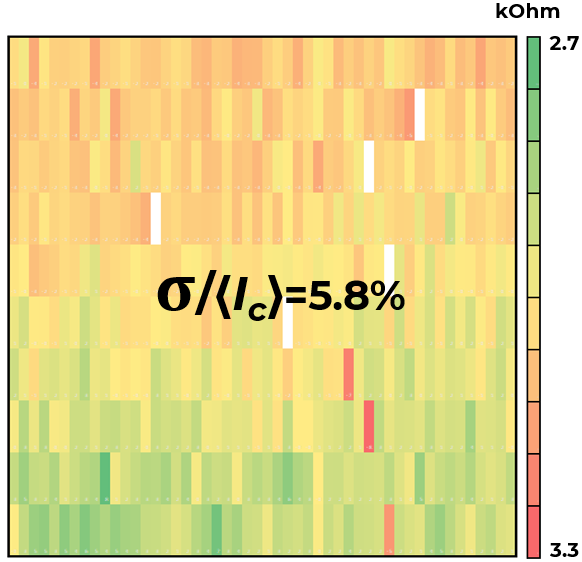 | 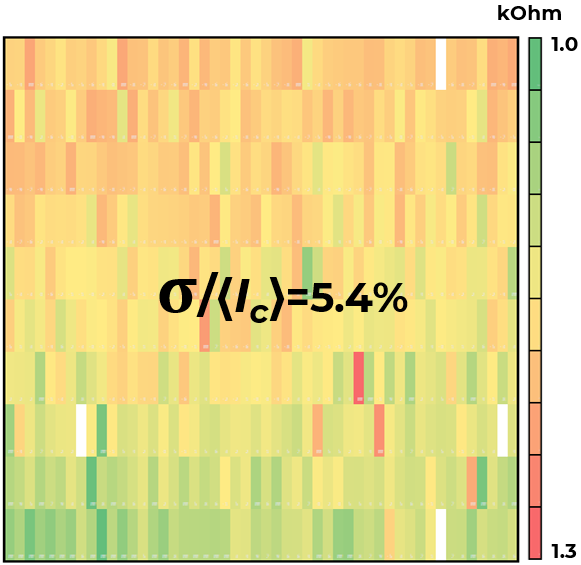 |
| **15 nm/0º** | 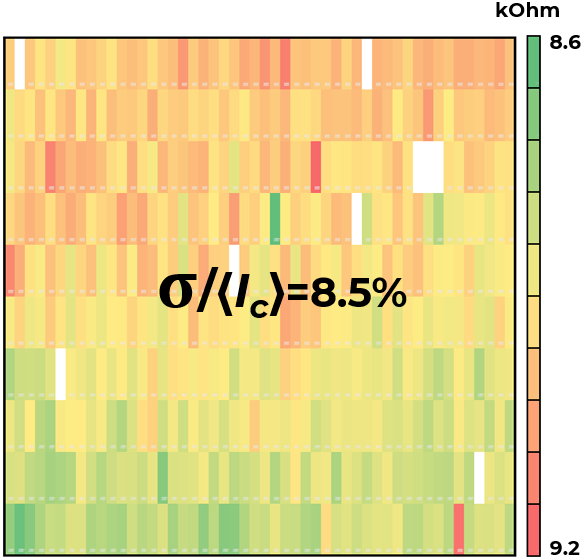 | 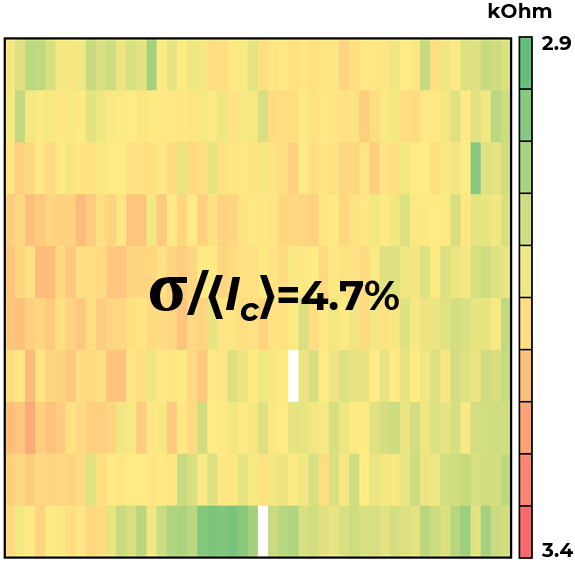 | 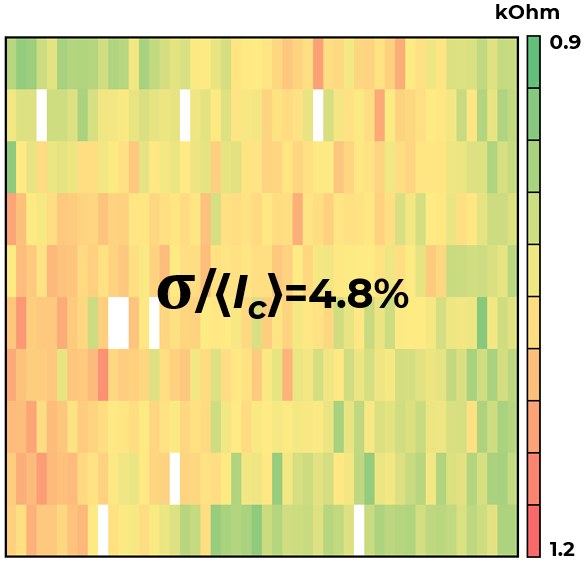 |

**Dynamic oxidation**

Experimentally found that transition to dynamic oxidation allowed reducing the spread of critical current over a chip for the oxygen pressures less than 0.1 mbar. We fabricated test chips with Josephson junctions, which were deposited in the optimal mode. Dynamic oxidation in pure oxygen at a pressure of 0.03 mbar was used. Critical current variation (*σ/⟨I_c_⟩*) of Josephson junctions for different areas (100×100 nm^2^, 150×200 nm^2^ and 150×600 nm^2^) is shown in Table S2.

**Table S2.** Critical current variation (*σ/⟨I_c_⟩*) of Josephson junctions for different areas (dynamic oxidation)

| **100×100 nm^2^ JJ area** | **150×200 nm^2^ JJ area** | **150×600 nm^2^ JJ area** |
| --- | --- | --- |
| 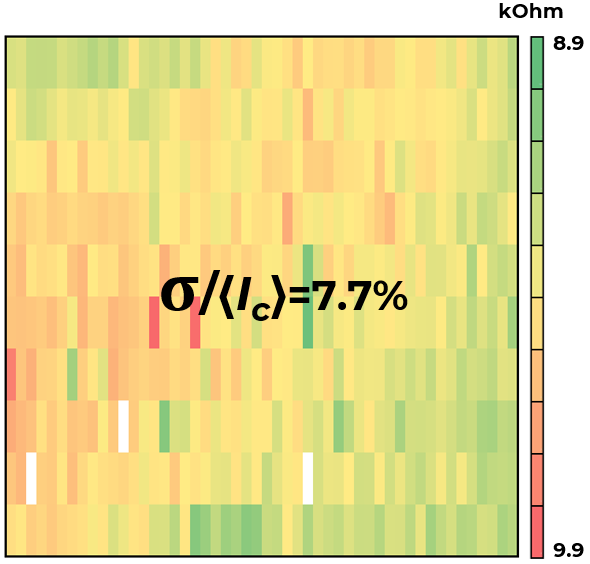 | 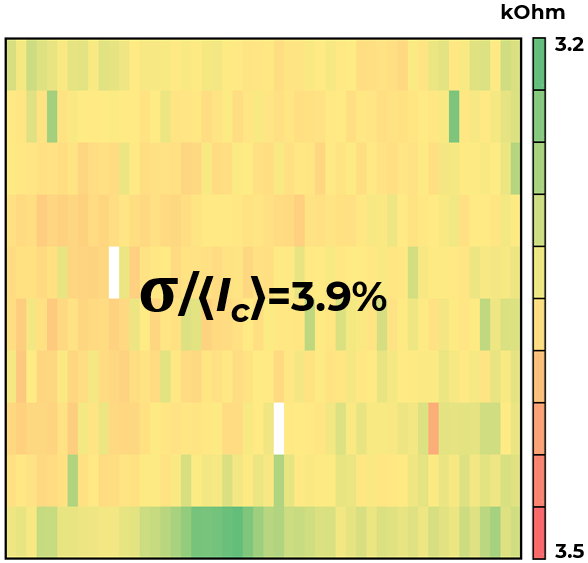 | 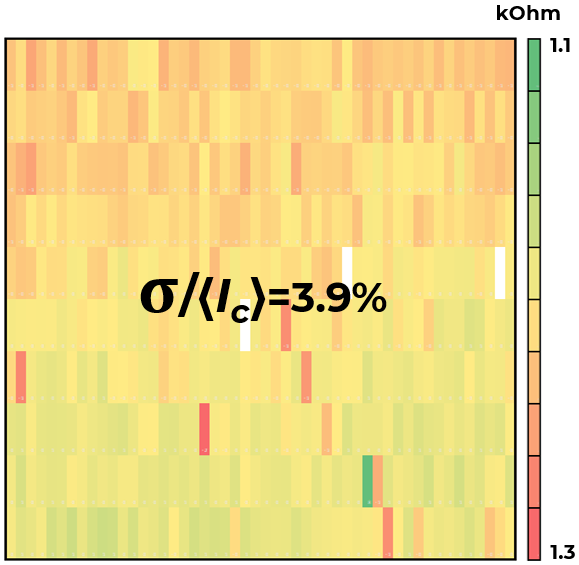 |

**Qubit measurements**

We fabricated three similar chips with 6 fixed-frequency transmon qubits on each to correlate observed improvements in the Josephson junction uniformity with ultimate device performance. Transmons were designed to a fixed frequency of 4.3 GHz and readout resonators with a frequency detuning between them of 100 MHz. The measurement results of one qubit from each chip are shown in Table S3.

**Table S3.** Coherence characterization for first qubits from each chip

| **Chip #1 (Qubit #1)** | 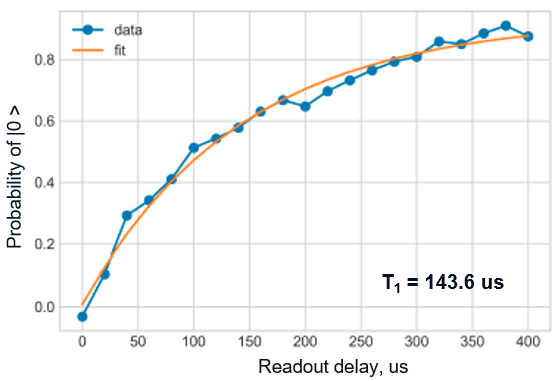 | 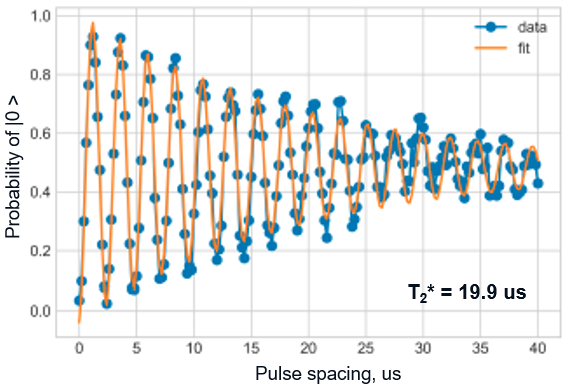 |
| --- | --- | --- |
| **Chip #2 (Qubit #1)** | 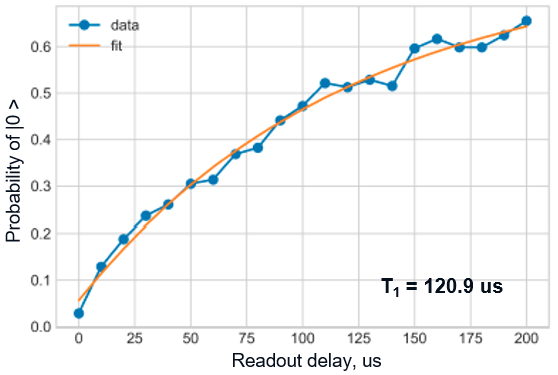 | 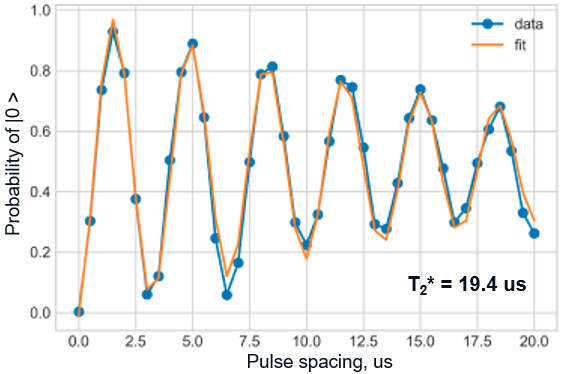 |
| **Chip #3 (Qubit #1)** | 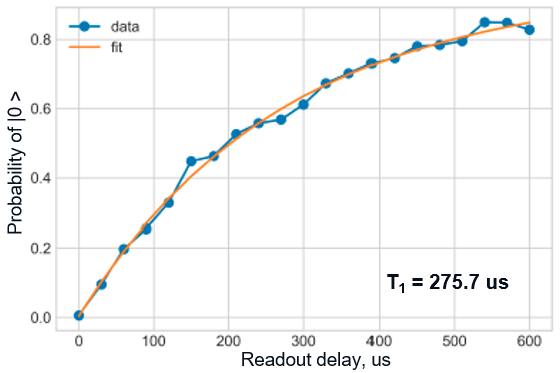 | 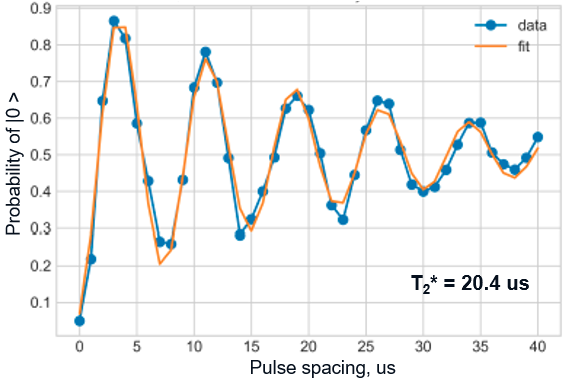 |

The image of single qubit on the chip is shown in Figure S1.


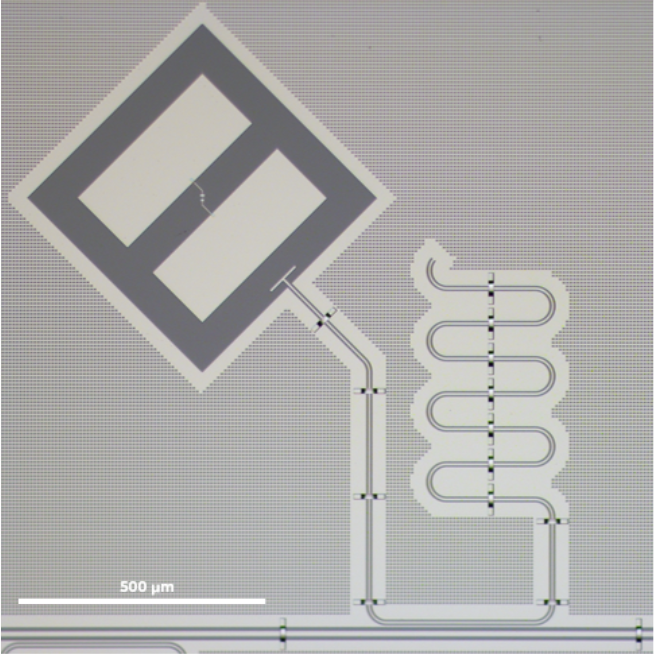


Figure S1. The optical image of a single qubit on a chip
